# Early complications in scoliosis surgery and relation to preoperative factors: A single-center retrospective study

**DOI:** 10.1097/MD.0000000000037529

**Published:** 2024-03-29

**Authors:** Hale Aksu, Busra Manduz, Mustafa Armagan, İsmail Safa Satoglu, Volkan Hanci

**Affiliations:** a Department of Anesthesiology and Reanimation, School of Medicine, Dokuz Eylul University, Izmir, Turkey; b Orthopedics and traumatology, School of Medicine, Dokuz Eylul University, Izmir, Turkey.

**Keywords:** anesthesia, risk factors, scoliosis

## Abstract

In this study, we aimed to investigate the perioperative complications of the patients who underwent scoliosis surgery in our hospital and the factors that may affect the outcome. Between 2014 and 2018, scoliosis patients recorded data was examined retrospectively. Age, gender, height, body weight, comorbidity, Cobb index, scoliosis etiology, operation time, preoperative and postoperative hemoglobin, hematocrit, leukocyte, blood urea nitrogen, creatinine, coagulation value, operation time, level of instrumentation, intraoperative and postoperative blood loss, blood transfusion, intraoperative fluid administration, preoperative pulmonary function test values, blood gas values, urine outputs, hospital (LOS) and post anesthesia care unit stays, complications and mortality rates were examined. The files of 77 patients (48 female, 29 male) were retrospectively analyzed. The average age was 19.54 ± 16.32 years and 98.7% were elective surgery. The mean of LOS was 13.55 ± 9.13 days. As the preoperative hematocrit value decreases, LOS increases significantly. In patients with chronic obstructive pulmonary disease, smokers and high ASA scores, LOS is prolonged in patients with previous operations. As intraoperative colloid administration increased, crystalloid and blood products increased, it was also observed that the amount of crystalloid increased LOS. As the amount of intraoperative colloid or red blood cell administration increases, the duration of surgery and anesthesia increases, also increases the duration of post anesthesia care unit. Compared to patients with complications (n = 29) to the patients without complications (n = 47), it was found that they had longer anesthesia, and surgery times, also longer LOS times (*P* < .05). Our study showed that chronic obstructive pulmonary disease in the preoperative period, smoking, high ASA score, excessive use of colloid, prolonged duration of surgery and anesthesia, and long intubation durations increase the length of hospital stay. Preoperative comorbidity is directly related to postoperative complications and causes longer hospitalization after reconstructive scoliosis surgery.

## 1. Introduction

The word scoliosis was first used by Galen and comes from the Greek word creeped (curved).^[1]^ This spinal deformity of the vertebrae, which is the most common and recognized since ancient times, was first described by Hippocrates; it is a multifactorial 3-dimensional structural anomaly of the spine characterized by anterior and posterior spinal growth anomalies, mostly with a female-male ratio of 8:1, seen in 3% of the general population.^[2]^ While the degree of curvature is constant in some, it increases over time.^[3]^ Mild scoliosis typically does not cause problems, but in more severe cases, it can affect breathing and movement.^[4,5]^ Pain is usually found in adults and may worsen with age. The purpose of surgery; to eliminate pain, pulmonary complications and psychological impact by removing the physical and cosmetic barrier created by the patient deformity. Corrective surgeries performed in scoliosis disorders are mostly surgeries with long surgical and anesthesia periods due to multiple vertebral involvement. Thoracic surgery affects lung volumes and oxygenation; anesthesia, pain and immobilization also contribute to this in the postoperative period.^[6,7]^ Administering high volumes of fluid and blood products may require close monitoring in the postoperative period due to the prolonged surgical time, the large number of vertebrae fused, and potential blood loss. For this reason, in patients who are planned for scoliosis surgery, preoperative evaluation, monitoring, knowing the changes in the respiratory and cardiovascular systems due to scoliosis, bleeding control, anesthesia method, hypothermia, fluid balance, neuromonitorization, pain control, postoperative care and problems gain importance.

Corrective surgeries performed in scoliosis disorders are mostly surgeries with long surgical and anesthesia periods due to multiple vertebral involvement. Due to the prolonged surgical time, the large number of vertebrae fused, and potential blood loss, administration of high volumes of fluid and blood products may require close monitoring in the postoperative period. In this study, we aimed to determine the risk factors affecting the need for intensive care by examining retrospective preoperative and intraoperative data in patients who underwent corrective scoliosis surgery and hospitalized in the post anesthesia care unit (PACU) between January 2014 and January 2018. Considering the preoperative and intraoperative risk factors, predetermining the patients who will need PACU will contribute to the management of intensive care resources. In addition, we aimed to determine the risk factors affecting the need for intensive care in order to prevent and solve the problems that may occur, to contribute to the current intensive care resource management, by retrospectively examining various parameters from the preoperative evaluation and preparation stage to the intraoperative and postoperative discharge period in patients undergoing scoliosis surgery.

## 2. Materials and methods

In our study, after local ethics committee approval (2020/24-09) was obtained, retrospective file analysis was performed in patients who underwent corrective scoliosis surgery and hospitalized in the PACU between January 2014 and January 2018, and it was aimed to determine the risk factors affecting the need for intensive care by examining the preoperative and intraoperative data. In the preoperative evaluation of the patients, age, gender, ASA (*American Society of Anesthesiologists*) class, body mass index, previous spine surgery, Cobb angle, scoliosis type, habits, systemic diseases (hypertension, atherosclerotic heart disease, arrhythmia, congestive heart failure, asthma, chronic obstructive pulmonary disease [COPD], cerebral palsy, epilepsy, muscular dystrophy). In the intraoperative period, anesthesia type (inhalation anesthesia, TIVA), operation time (anesthesia time, surgery time, extubation time), start and end time of the operation, size of the surgery (number of vertebrae fused, above T5, between T5 and T8, below T8, lumbar spine), amount of blood loss, crystalloid-colloid volume administered, blood volume administered, neuromonitoring application, body temperature, antifibrinolytic use, urine amount (mL/h), arterial blood gas abnormalities were investigated. In the postoperative evaluation, airway maintenance, PACU transport, reason for transport (hypotension, acidosis, comorbidity, monitoring, hypothermia, other reasons), extubation time, postoperative pain control, postoperative early or late complications, duration of stay in the PACU, postoperative hospital stay (LOS) was evaluated from hospital records and patient files. The extubation time was obtained by calculating the postanesthetic end time, in hours, from which the patient was extubated. Patients who were already intubated, patients with tracheostomy, patients who were planned to be intubated after the first surgery when a 2-step procedure was planned, and patients with missing data in the registration form were excluded from the study.

In our study, we aimed to determine the risk factors affecting the need for intensive care in order to prevent and solve the problems that may occur, and to contribute to the current intensive care resource management, by retrospectively examining various parameters starting from the preoperative evaluation and preparation stage to the intraoperative and postoperative discharge period in patients who have undergone scoliosis surgery. Considering the preoperative and intraoperative risk factors, predetermining the patients who will need PACU contributes to the management of intensive care resources.

### 2.1. Statistical analysis

In the descriptive statistics of the data, mean, standard deviation, median minimum, maximum, frequency and ratio values were used. The distribution of variables was measured with the Kolmogorov Smirnov test, and the Mann–Whitney *U* test and independent sample *t* test were used in the analysis of quantitative data. Chi-square test was used in the analysis of qualitative data, and Fischer test was used when the chi-square test conditions were not met. Correlation analyzes were performed with the Pearson correlation analyzes SPSS 22.0 program was accepted as a significant value of *P* < .05 in the analyses.

## 3. Results

When the data were compiled, a total of 77 cases who underwent corrective scoliosis surgery and were hospitalized in the PACU were found. The ages of the patients; the smallest 3 and the largest 80 (mean 19.54 ± 16.32), gender distribution; 48 (62.3%) were female and 29 (37.7%) were male. Considering the age distributions by gender; It was seen that 48 female subjects (mean 19.60 ± 16.64) and 29 male subjects (mean 19.44 ± 16.05) had mean age distributions. One (1.3%) of the 77 cases who were operated on was taken to the operation as an emergency, and it was observed that 76 (98.7%) cases were operated under elective conditions. When the ASA scores of the patients were examined; It was determined that 21 (27.6%) cases were ASA I, 47 (61.8%) cases were ASA II, and 8 (10.5%) cases were ASA III. The distribution of preoperative and postoperative laboratory data is shown in Table 1.

**Table 1:** The distribution of preoperative and postoperative laboratory data.

Laboratory parameter	Preoperative value	Postoperative value	P
Leukocytes (10*3/µL)	7.53 ± 1.98	15.06 ± 5.39	<.001
Hct (%)	38.07 ± 4.76	29.44 ± 4.35	<.001
BUN (mg/dL)	12.47 ± 4.95	11.69 ± 6.23	.339
Creatinine (mg/dL)	0.52 ± 0.21	0.45 ± 0.22	<.001
Bilirubin (mg/dL)	0.92 ± 2.17	1.09 ± 0.69	.562
AST (U/L)	23.76 ± 8.63	49.47 ± 46.35	<.001
ALT (U/L)	16.74 ± 8.62	22.08 ± 27.98	.087
INR	1.06 ± 0.07	1.33 ± 0.27	<.001

Mean ± SD; Mann–Whitney *U* test.

ALT = alanine aminotransferase, AST = aspartate aminotransferase, BUN = blood urea nitrogen, Htc = hematocrit, INR = international normalized ratio.

In the Pearson correlation analysis; It was determined that there was a weak positive correlation between preoperative Hct (*r* = −0.261; *P* = .026) and length of hospital stay. When all the cases are examined; The mean length of hospital stay was (13.55 ± 9.13). The distribution of habits and systemic diseases and their effects on the length of hospital stay are examined in Table 2.

**Table 2:** Effects of habits and systemic diseases on hospital stay.

	n (%)	r	P
Smoking	6 (7.9)	0.268	.021*
Lung Infection	3 (4.0)	−0.039	.745
Wound Infection	6 (8.0)	0.034	.007*
Systemic Infection	3 (3.9)	0.091	.441
Pneumo/Hemothorax	2 (2.6)	0.045	.704
Reoperation Hypertension Diabetes COPD Chronic renal failure ASA	21 (27.6) 6 (7.9) 2 (2.6) 1 (1.3) 1 (1.3) 41 (54.7) ASA I 21 (27.6) ASA II 47 (61.8) ASA III 8 (10.5)	0.206 0.227 0.008 0.238 −0.007 0.259 0.405	.078 .051 .945 .041* .952 .027* <.001*

*Bold values were found to be statistically significant.

COPD = chronic obstructive pulmonary disease.

In the Pearson correlation analysis; It was observed that the duration of hospitalization was significantly longer in patients with smoking, presence of wound infection, COPD, previous operation history, and patients with high ASA scores.

According to this; It was determined that as the duration of anesthesia and surgery increased, the duration of hospitalization increased. It was observed that the duration of hospitalization increased significantly as the amount of postoperative crystalloid increased. It was found that as the perioperative transamine dose increased, there was a significant increase in intraoperative crystalloid, postoperative day 1 (POD1) urine, surgery time and anesthesia time. As the amount of intraoperative colloid use increases; It was observed that the amount of intraoperative crystalloid use, the amount of red blood cell (RBC) and the amount of other blood products increased, as well as POD1 urine, surgery time, anesthesia time and intubated time increased (Tables 3 and 4).

**Tablo 3:** Intraoperative factors affect on length of stay.

Intraoperative factor	Mean ± SD	r	P
Transamine dose n = 58 (77.3%)	596.71 ± 454.26	−0.007	.955
Intraoperative colloid	965.13 ± 852.50	−0.119	.315
Intraoperative crystalloid	3408.10 ± 1665.05	−0.081	.497
RBC amount	2.29 ± 1.75	0.101	.393
Other blood product amount	1.07 ± 1.44	0.000	.998

RBC = red blood cell.

**Tablo 4:** Postoperative factors affect on length of stay.

Postoperative factor	Mean ± SD	r	P
POD1 urine	1005.00 ± 900.25	−0.34	.774
Postoperative crystalloid	660.48 ± 442.82	0.254	.037*
Amount of postoperative RBC	0.75 ± 0.99	0.179	.139
POD2 urine	579.14 ± 385.55	0.329	.006*
Surgical Time	6.04 ± 1.77	0.242	.040*
Anesthesia duration	6.90 ± 1.89	0.237	.045*
Intubation time	11.61 ± 3.76	0.325	.006*

*Bold values were found to be statistically significant.

POD1 = postoperative day 1, POD2 = postoperative day 2, RBC = red blood cell.

Considering the duration of stay in the postanesthetic care unit and intensive care unit; It was found that patients stayed in PACU for a mean of (19.40 ± 6.92) hours and in ICU for a mean (1.33 ± 0.57) days, 94.7% of all patients were in PACU and 5.3% It was revealed that he was in the ICU.

When the data were analyzed in detail, it was determined that there was a positive correlation between the duration of PACU hospitalization (*r* = 0.489; *P* < .001) and the duration of hospitalization. In addition, PACU hospitalization; amount of intraoperative colloid (*r* = 0.266; *P* = .032), amount of intraoperative RBC (*r* = 0.276; *P* = .025), amount of postoperative crystalloid (*r* = 0.306; *P* = .014), amount of postoperative RBC (*r* = 0.304; *P* = .013), postoperative day 2 urine volume (*r* = 0.414; *P* = .001), duration of surgical operation (*r* = 0.357; *P* = .004), duration of anesthesia (*r* = 0.360; *P* = .003), and intubated follow-up time (*r* = 0.603; *P* = .003) 0.000 and there is a positive correlation between them.

When the relationship between preoperative and postoperative laboratory tests and the duration of PACU hospitalization was examined, it was observed that as the Hct levels measured preoperatively, the PACU hospitalization was shorter (*r* = −0.358; *P* = .003), but there was no significant relationship between them and other laboratory parameters.

When the distribution of habits and systemic diseases and their effects on the duration of stay in PACU are examined; It was revealed that in patients with a previous operation (*r* = 0.348; *P* = .005), the length of PACU stay was prolonged and as the ASA score of the patients increased (*r* = 0.329; *P* = .007), there was a significant prolongation of the duration of PACU hospitalization. It was observed that the patients had preoperative habits and comorbidities such as smoking, COPD, and prolonged LOS. On the other hand, factors such as lung infection, wound infection, presence of systemic infection, hemo/pneumothorax, hypertension, diabetes, chronic renal failure did not have a significant effect on the duration of PACU hospitalization.

The incidence of postoperative complications and their effect on the duration of PACU hospitalization are shown in Table 5.

**Table 5:** Relationship between postoperative complications and PACU length of stay.

Postoperative complication	n (%)	r	P
postoperative early/late neurological deficit	4 (5.2)	0.288*	.038*
Postoperative paraplegia	2 (2.6)	0.200	.156
Postoperative radiculopathy	4 (5.2)	0.027	.853
Other complications (wound site inf, pleural eff, etc)	15 (19.5)	0.329*	.017*

*Bold values were found to be statistically significant.

PACU = post anesthesia care unit.

In the Pearson correlation analysis; It was observed that postoperative early/late neurological deficits and other complications such as wound infection, pleural effusion were significantly correlated with the length of stay in PACU, and there was a positive correlation, thus prolonging the duration of PACU hospitalization.

The correlation analysis of the factors that have a significant effect on the duration of PACU stay is given in Table 6.

**Table 6:** Correlation analysis of factors affecting PACU length of stay.

	Preoperative neurological finding	Postop neurological deficit	Postop paraplegia	Postop radiculopathy	Other complications
PACU hospitalization time	**0.352** *	**0.288** *	0.200	0.027	**0.329** *
Preop BUN	0.159	**0.279** *	−0.035	−0.151	−0.108
Preop AST	−0.098	−0.066	−0.189	−0.136	**−0.361** **
Preop INR	−0.113	−0.296*	0.159	−0.039	−0.059
Postop HCT	−0.166	0.030	**−0.287** (*)	−0.059	−0.100
Lung infection	**0.281** *	−0.052	−0.036	−0.053	**0.395** **
Wound infection	**0.404** **	0.193	**0.321** *	0.192	**0.461** **
Systemic infection	0.197	−0.037	−0.025	−0.037	**0.320** *
Pneumo/Hemothorax	−0.130	−0.052	−0.036	−0.053	**0.320** *
Reoperation	**0.416** **	**0.440** **	**0.305** *	**0.302** *	**0.428** **
COPD	0.197	−0.037	**0.701** **	–	0.224
Previous operation	**0.334** *	0.258	0.179	0.265	**0.307** *
Smoking	**0.323** *	**0.400** **	**0.278** *	**0.462** **	0.239
ASA	0.188	0.211	0.223	0.115	0.023
Preoperative neurological finding	–	**0.404** **	**0.281** *	0.118	**0.401** **
Postop neurological deficit	**0.404** **	–	−0.052	0.192	**0.322** *
Postop paraplegia	**0.281** *	−0.052	–	**0.486** **	**0.334** *
Postop radiculopathy	0.118	0.192	**0.486** **	–	0.176
Other complications	0.401**	**0.322** *	**0.334** *	0.176	–

Bold values were found to be statistically significant.

Pearson correlation analysis.

* *P* < .05.

** *P* < .01.

AST = aspartate aminotransferase, BUN = blood urea nitrogen, COPD = chronic obstructive pulmonary disease, INR = international normalized ratio, PACU = post anesthesia care unit.

## 4. Discussion

Our study showed that COPD in the preoperative period, smoking, high ASA score, excessive use of colloid, prolonged duration of surgery and anesthesia, and long intubation durations increase the length of hospital stay.

Blood urea nitrogen, international normalized ratio and hematocrit values in the preoperative period, neurological sequelae in the preoperative or postoperative period, pulmonary, wound or systemic infection in the postoperative period, intraoperative pneumonia/hemothorax development in the patient, increase in POD1 urine output and complications in the postoperative period are related to PACU and LOS durations, shows a significant correlation.

Clinical signs, symptoms, multifactorial conditions in its pathophysiology, surgical procedure and possible postoperative complications; This has made scoliosis surgery one of the most challenging steps of spinal surgery in terms of anesthetic management. Patients undergoing spinal fusion and instrumentation for scoliosis may experience a high rate of postoperative complications that may require admission to the intensive care unit and the need for mechanical ventilation. While not the sole focus, shortening the length of stay is particularly important as an outcome criterion and contributes to improving hospital efficiency and reducing costs. Length of stay is a complementary measure of better care and reduces potential medical errors.^[8,9]^ In our study, the data of 77 patients were scanned retrospectively, the gender distribution was male/female 29/48, the mean age was 19.54 (±16.32) years, the length of hospital stay was 13.55 (±9.13) days, and the length of hospitalization was found to be significantly longer as the preoperative hematocrit decreased. In a study conducted by Guan et al^[10]^ to determine whether the preoperative Hct level has a significant effect on hospitalization in patients undergoing routine lumbar spinal procedures, preoperative anemia was indeed associated with longer hospital stay, and the mean LOS was 1 day (%) in patients with preoperative anemia. 30 showed that it was longer. Again, in our study, we found that there was a significant decrease in the duration of PACU hospitalization as the preoperative Hct increased. Anemia has been shown to be an indicator of poor outcome in many surgical procedures.^[11,12]^ With the high blood loss often associated with spinal procedures, it is plausible that this population is particularly vulnerable to baseline anemia.^[13]^

In our study, it was revealed that smoking, presence of COPD and high ASA score were associated with prolonged hospital stay. Zirka et al^[14]^ found that age and ASA level were significant in their study investigating factors associated with delayed extubation after multilevel spine surgery. In this respect, our study is compatible with the literature. In patients with COPD and smokers, we also observed that the length of hospital stay was prolonged. Zhang et al^[15]^ in his study; In this study, 298 cases who underwent scoliosis surgery were evaluated with pulmonary function test before the operation, and the possible complications expected to be seen postoperatively (atelectasis, hydrothorax, pneumothorax, pneumonia, hypoxemia, postoperative mechanical ventilation need) were evaluated. The incidence of postoperative complications was found to be higher in cases with preoperative pulmonary symptoms and abnormal pulmonary function test values. In another study conducted by Adogwa et al^[16]^ to investigate the effect of postoperative complications and patient comorbidities on the variability in the prolongation of hospital stay after lumbar spine surgery, it was observed that COPD prolongs LOS. In this respect, the results of our study are compatible with the literature.

In our study, we found that patients with wound infection and previous operation had a significantly longer hospital stay. The incidence of wound infections in scoliosis surgery is 1% to 3%.^[17–19]^ Therefore, it can be considered as a rare but important complication. Important predictors of increased LOS in adolescent idiopathic scoliosis patients undergoing posterior spinal fusion were in-hospital complications, with wound-related complications being the strongest predictor, and patients with wound-related complications had an increased LOS compared to those without wound complications. Yoshiara et al^[20]^ this were 3.14 times more likely to have.

When the intraoperative data were evaluated in our study, no correlation was found between the use and dose of tranexamic acid (TXA) and the duration of hospitalization; we have seen that there is an increase in the use of intraoperative crystalloid with the use and dose of TXA, the duration of surgery and accordingly the time spent under anesthesia, the time spent intubated increases, and the amount of POD1 urine increases. It can be thought that additional doses applied other than our routine TXA dose regimen in scoliosis surgeries are requested by the surgical team, possibly due to bleeding in the surgical field. We think that the surgical time is prolonged due to the intervention of the bleeding focus and the long duration of bleeding control by the team. Prolonged surgical time; may result in additional blood losses, increased fluid administration, and increased postoperative blood transfusion. Prolonged operative time has been shown to be an independent risk factor for postoperative complications in spine surgery.^[21]^ There are many factors affecting the surgical time; The experience of the surgeon and operating room team may also be affected by the stiffness of the curve, the patient height, the difficulty of implant placement, and the proficiency of the radiology technician who obtains intraoperative radiographs with fluoroscopic images.^[22]^ Spinal surgery is often associated with massive blood loss that requires blood transfusion. Antifibrinolytic TXA is a lysine analog that inhibits the activation of plasminogen and is known to be useful in reducing surgical blood loss. In a meta-analysis of 11 randomized controlled trials, Cherian et al^[23]^ showed that TXA reduced intraoperative, postoperative, and total blood loss by an average of 219 mL, resulting in a reduction in the proportion of patients requiring blood transfusion compared to patients receiving placebo. In our study, it can be said that there was no increase in the use of blood transfusion products in cases where the use of TXA increased.

We have seen that with the increase in the use of intraoperative colloids, there is an increase in the use of crystalloids and blood products, the duration of surgery and, accordingly, the time spent under anesthesia, the increase in the time spent intubated, and the increase in the amount of POD1 urine. Here, too, we think that the anesthetist is trying to stabilize the patient vital values and minimize the use of blood products by increasing the amount of colloid before giving the blood product. It was observed that the amount of crystalloid increased the LOS as the intraoperative colloid application increased and the crystalloid and blood products increased. In deformity surgery, the amount of bleeding from the surgical area is high; blood loss is approximately 1/3 of the total blood volume in uncomplicated cases, and this amount is even higher in neuromuscular scoliosis.^[24]^ If blood loss and other causes (such as spinal shock) cause hemodynamic changes, the amount of crystalloid, colloid and blood used also increases. In a study conducted to determine the factors affecting the length of stay (LOS) in patients who underwent minimally invasive lumbar instrumentation between 2008 and 2010, cases with a prolonged hospital stay had significantly higher estimated blood loss, the patient received more crystalloids, had higher total fluid, longer surgical time, lower end-of-case temperature, lower hemoglobin during hospitalization.^[25]^ In the same study, important markers of increased LOS were shown to be postoperative creatinine, intraoperative colloids, fluid entry at the end of the surgical case and crystalloid/colloid ratio, mean FiO_2_, and preoperative hemoglobin level.^[25]^ When all these data are examined, our study seems to be compatible with the literature.

In our study, it was aimed to determine the risk factors affecting the need for paw/intensive care by examining the preoperative and intraoperative data. As the amount of intraoperative colloid increases, the amount of erythrocyte suspension increases, and the amount of postoperative crystalloid and postoperative erythrocyte suspension increases, the duration of PACU stays longer; In addition, it was revealed that as the duration of surgery increases, the duration of PACU increases as the duration of anesthesia and intubation increase. Li et al^[26]^ showed that intraoperative factors such as prolonged surgery time, significant blood loss, large amount of crystalloid-colloid infusion and blood transfusion may be risk factors for prolonged extubation after thoracic and lumbar surgery. Again, in our study, it was revealed that the PACU stay was prolonged in patients with a previous operation, and as the ASA score of the patients increased, there was a significant prolongation of the PACU hospitalization period. On the other hand, factors such as smoking, lung infection, wound infection, presence of systemic infection, hemo/pneumothorax, HT, DM, COPD, chronic renal failure did not have a significant effect on the duration of PACU hospitalization. In a study by Yerebakan et al,^[27]^ they demonstrated a significant increase in the need for intensive care in patients with ASA level II and above, which is one of the preoperative factors. Kay et al^[28]^ emphasized that age, female gender, ASA score, cardiac comorbidities, intraoperative blood loss, and duration of surgery were significantly associated with postoperative ICU stay.

When the postoperative complications are examined; It was determined that patients with complications (n = 29) had longer durations of anesthesia and surgery than patients without complications (n = 47), and also had longer hospital stays. Again, in the correlation analysis; Postoperative early/late neurological deficits and other complications such as wound infection, pleural effusion were observed to prolong the duration of PACU hospitalization. Pulmonary complications are the most important cause of mortality and morbidity after surgical intervention, and the incidence in non-idiopathic scoliosis is 5 times higher than in idiopathic scoliosis.^[29]^ The frequency is higher in anterior approaches than in posterior approaches. Atelectasis, infiltrates, hemothorax, pneumothorax, pleural effusion and prolonged intubation time are common problems; pneumonia, pulmonary edema, and upper airway obstruction are less common.^[24]^ The limitations of our study include the lack of long-term follow-up of the patients and the fact that it was conducted only in the patient population taken to the post-operative post-anesthesia care unit.

## 5. Conclusion

The preoperative, intraoperative, and postoperative factors identified in this study should aid surgical planning and prevent complications to reduce health care costs associated with prolonged hospital stay. Preoperative comorbidity is directly related to postoperative complications and causes a longer hospital stay after corrective scoliosis surgery. Ensuring optimal hemoglobin levels prior to surgery will reduce hospital stay. Intraoperative management of scoliosis is also an important factor affecting the length of stay in the PACU.

## Author contributions

**Conceptualization:** Hale Aksu, Ismail Safa Satoglu.

**Data curation:** Busra Manduz, Mustafa Armagan, Ismail Safa Satoglu, Volkan Hanci.

**Formal analysis:** Hale Aksu, Volkan Hanci.

**Funding acquisition:** Hale Aksu.

**Investigation:** Hale Aksu, Ismail Safa Satoglu.

**Methodology:** Hale Aksu, Mustafa Armagan, Volkan Hanci.

**Resources:** Hale Aksu.

**Supervision:** Hale Aksu, Volkan Hanci.

**Writing – original draft:** Hale Aksu, Busra Manduz, Volkan Hanci.

**Writing – review & editing:** Hale Aksu, Volkan Hanci.
